# Patient-Reported Quality of Life and Functional Impairment in Liver Cirrhosis: Clinical Determinants from a Cross-Sectional Study

**DOI:** 10.3390/healthcare14193154

**Published:** 2026-09-23

**Authors:** Roxana Elena Mirică, Raluca Mesteru, Claudia Simona Stefan, Alina Breazu, Laura Maria Batis, Ioana Soare

**Affiliations:** 1 Faculty of Medicine, “Carol Davila” University of Medicine and Pharmacy, 020021 Bucharest, Romania; 2 Regina Maria Private Healthcare Network, Policlinica Floreasca, 011736 Bucharest, Romania; 3 National Institute of Medical Expertise and Work Capacity Recovery (INEMRCM), 050659 Bucharest, Romania; rra.mesteru@gmail.com; 4 Faculty of Medicine and Pharmacology, “Dunarea de Jos” University, 800008 Galati, Romania; claudia.stefan2009@ugal.ro; 5 Public Health and Health Services Management, National Institute of Health Services Management, 021253 Bucharest, Romania; dr_alinabreazu@yahoo.com; 6 Department of Geriatrics, Faculty of Medicine, “Titu Maiorescu” University, 040051 Bucharest, Romania; laurabatis@yahoo.com; 7 Insurance Medicine Department, “Titu Maiorescu” University, 040051 Bucharest, Romania; ioana_soare@yahoo.com

**Keywords:** liver cirrhosis, health-related quality of life, Child–Pugh classification, patient-reported outcomes, functional impairment, social isolation

## Abstract

**Background:** Liver cirrhosis imposes a substantial clinical, functional, and psychosocial burden, significantly affecting patients’ health-related quality of life (HRQoL). The aim of this study was to evaluate the relationship between disease severity, functional status, and HRQoL in patients with liver cirrhosis. **Methods**: This cross-sectional observational study included 144 adult patients with liver cirrhosis of diverse etiologies. Clinical, demographic, and laboratory data were collected, while HRQoL was assessed using the Short Form Liver Disease Quality of Life (SF-LDQOL) questionnaire. Disease severity was evaluated according to the Child–Pugh classification. Statistical analysis included descriptive statistics, Spearman correlation, the Kruskal–Wallis test, and Firth penalized multivariable logistic regression. **Results**: Most patients were classified as Child–Pugh A (75.7%), followed by Child–Pugh B (17.4%) and Child–Pugh C (6.9%). Overall quality of life was predominantly rated as fair or poor. Greater liver-related symptom burden and limitations in daily activities were significantly associated with poorer patient-reported quality-of-life outcomes. Overall quality of life differed significantly across Child–Pugh classes (*p* < 0.001), with poorer ratings in Child–Pugh B and C than in Child–Pugh A, although impaired quality of life was also observed among patients with compensated cirrhosis. In the forced-entry multivariable clinical model, Child–Pugh B was significantly associated with higher odds of impaired overall quality of life compared with Child–Pugh A (adjusted OR 33.32, 95% CI 1.90–582.94, *p* = 0.016), whereas age, sex, and etiology were not independently associated with the outcome. **Conclusions**: Health-related quality of life in patients with liver cirrhosis is associated with disease severity as well as with functional and psychosocial aspects of the patient-reported disease experience. Integrating patient-reported outcome measures with conventional clinical assessment may provide a more comprehensive evaluation of disease burden and support individualized multidisciplinary patient management.

## 1. Introduction

Liver cirrhosis represents one of the most common causes of advanced chronic liver disease, with a significant impact on morbidity, mortality, and patients’ quality of life [1,2]. Among its etiologies, chronic alcohol consumption remains a major contributor, leading to progressive structural and functional hepatic changes, frequently associated with systemic complications and overall deterioration of health status [3].

The global burden of liver cirrhosis continues to increase, representing a major public health challenge due to its high rates of hospitalization, disability, and premature mortality. Beyond its impact on healthcare systems, cirrhosis is associated with substantial socioeconomic consequences due to frequent hospitalizations, long-term medical care, and impaired daily functioning. Consequently, preserving functional status and improving health-related quality of life have become important therapeutic goals alongside the prevention of disease progression and liver-related complications [4,5]. Chronic liver disease encompasses a broad spectrum of progressive hepatic disorders that may evolve from compensated disease to decompensated cirrhosis and major complications, including hepatocellular carcinoma. This broader clinical continuum highlights the heterogeneous biological and clinical burden of chronic liver disease and the need for multidimensional approaches that consider not only disease progression and complications but also their wider impact on patients’ health and functioning.

Beyond clinical progression and survival, patients with liver cirrhosis often experience important functional limitations, including reduced physical capacity, fatigue, cognitive impairment, emotional distress, and decreased social participation. These manifestations substantially affect patients’ health-related quality of life and overall functional status, emphasizing the need for a comprehensive evaluation that extends beyond conventional measures of disease severity [6,7].

In recent years, increasing attention has been directed toward patient-reported outcomes as complementary measures of disease burden in chronic liver disease. Health-related quality of life has emerged as a clinically relevant endpoint because it reflects the patient’s perception of physical functioning, emotional well-being, cognitive performance, and social participation. Unlike conventional prognostic scores, patient-reported outcome measures provide valuable information regarding the functional and psychosocial consequences of liver cirrhosis and may facilitate a more comprehensive assessment of disease burden and individualized patient management [8].

Previous studies have shown that patients with liver cirrhosis present with significantly impaired health-related quality of life, which tends to worsen with increasing disease severity and the presence of complications [6,9]. However, several reports suggest that subjective perception of health status may not always correlate with objective clinical parameters, highlighting the complexity of patient evaluation in chronic liver disease [6].

The assessment of health-related quality of life in patients with liver cirrhosis requires an integrated approach, as conventional measures of disease severity do not fully capture the functional and psychosocial burden experienced by patients. Patient-reported outcome measures complement conventional clinical assessment by reflecting the impact of liver disease on daily functioning, symptom burden, and psychosocial well-being.

The present study aimed to evaluate health-related quality of life in patients with liver cirrhosis using patient-reported outcome measures and to investigate its relationship with disease severity, symptom burden, functional limitations, and psychosocial factors. In addition, we explored the independent factors associated with impaired health-related quality of life in order to better characterize the multidimensional impact of liver cirrhosis on patients’ daily functioning.

## 2. Materials and Methods

### 2.1. Study Design

This observational cross-sectional study was conducted at the National Institute of Medical Expertise and Work Capacity Recovery, Bucharest, Romania, and included a consecutive cohort of adult patients with liver cirrhosis evaluated over a one-year period. The study was approved by the Ethics Committee of the National Institute of Medical Expertise and Work Capacity Recovery, Bucharest, Romania.

### 2.2. Study Population

A total of 146 adult patients with a confirmed diagnosis of liver cirrhosis were eligible for inclusion during the study period. Of these, 144 patients completed the SF-LDQOL questionnaire and were included in the final analysis, while 2 patients were non-responders. No missing items were identified among the completed questionnaires. The diagnosis of cirrhosis was established based on clinical, biochemical, and imaging findings. Patients with liver cirrhosis of diverse etiologies were included and analyzed as a mixed-etiology cohort; alcohol-related cirrhosis represented the most frequent individual etiology.

### 2.3. Inclusion Criteria

- age over 18 years
- confirmed diagnosis of liver cirrhosis, regardless of etiology
- provision of informed consent for participation and questionnaire completion

### 2.4. Exclusion Criteria

- presence of severe comorbid conditions with an independent impact on functional status or quality of life
- inability to complete the questionnaire due to cognitive or communication impairment

### 2.5. Data Collection

For each patient, the following data were collected:

- demographic data: age, sex, area of residence
- clinical data: reported symptoms and relevant findings from the clinical examination
- paraclinical data, obtained from medical records
- parameters required for liver disease severity classification, according to the Child–Pugh score
- patient-reported outcomes, including health-related quality of life, symptom burden and functional limitations

The questionnaire was self-administered at the time of clinical evaluation.

### 2.6. Assessment of Liver Disease Severity

The severity of liver cirrhosis was assessed using the Child–Pugh classification, based on five parameters: serum bilirubin, serum albumin, prothrombin time/INR, ascites, and hepatic encephalopathy. Each parameter was assigned 1–3 points, with total scores of 5–6 corresponding to Child–Pugh class A, 7–9 to class B, and 10–15 to class C. Patients were categorized accordingly.

### 2.7. Assessment of Health-Related Quality of Life

The impact of liver disease on functional status and health-related quality of life was evaluated using the SF-LDQOL questionnaire, which explores multiple domains including physical, emotional, social, and functional well-being [9,10]. The SF-LDQOL questionnaire was selected because it combines the generic assessment of health-related quality of life with disease-specific domains relevant to chronic liver disease. In addition to physical functioning and general health perception, the instrument evaluates fatigue, emotional well-being, social functioning, cognitive performance, disease-related concerns, and the impact of liver disease on daily activities [11]. This multidimensional instrument provides a comprehensive assessment of patients’ perceived health status and has been extensively used in studies evaluating health-related quality of life in chronic liver disease. The original development and validation work supports the reliability and validity of disease-specific quality-of-life assessment in patients with chronic liver disease [9,10]. Social functioning was assessed as one of the broader health-related quality-of-life domains, whereas social isolation was operationalized separately using the five-item loneliness component of the SF-LDQOL questionnaire. The items address perceived lack of companionship, lack of someone with whom to communicate, feeling abandoned, feeling isolated from others, and the ability to find companionship when desired. Responses were coded on an ordinal 0–4 scale (0 = never, 1 = rarely, 2 = sometimes, 3 = often, 4 = always), with higher scores indicating greater social isolation.

Limitations in daily activities and liver-related symptom burden were likewise operationalized as ordinal patient-reported measures on a 0–4 scale, with higher scores indicating greater functional limitation or symptom burden, respectively. The response categories were coded as 0 = none, 1 = slight, 2 = moderate, 3 = much, and 4 = very much. These ordinal measures were used in the descriptive and correlation analyses to characterize functional, symptomatic, and psychosocial aspects of the patient-reported disease experience. They were not included as predictors in the revised primary multivariable clinical model in order to avoid conceptual overlap with the overall quality-of-life outcome.

For the purposes of the present study, the SF-LDQOL was used as a multidimensional patient-reported outcome measure, and no single total SF-LDQOL score was calculated. Overall quality of life was assessed using the general health perception item. For the primary multivariable analysis, responses were categorized as preserved quality of life (excellent/good) versus impaired quality of life (fair/poor). The individual questionnaire domains and items were used to characterize specific aspects of patients’ health-related quality of life and functional and psychosocial status.

### 2.8. Statistical Analysis

Data were analyzed using descriptive and inferential statistical methods. Continuous variables are presented as mean ± standard deviation (SD), while categorical variables are expressed as absolute numbers and percentages.

Associations between clinical variables, functional limitations, and patient-reported quality-of-life domains were assessed using Spearman’s rank correlation coefficient, as several questionnaire variables were measured on ordinal scales and did not meet the assumptions for parametric testing. Differences in overall quality of life across Child–Pugh classes were evaluated using the Kruskal–Wallis test. When the overall test was statistically significant, pairwise comparisons between Child–Pugh classes were performed using Dunn’s posthoc test with Bonferroni adjustment for multiple comparisons. In addition, the relationship between Child–Pugh class and overall quality of life was evaluated separately in alcohol-related and non-alcohol-related cirrhosis subgroups using the Kruskal–Wallis test.

To identify clinical and demographic factors independently associated with impaired overall quality of life, a forced-entry multivariable logistic regression model was specified a priori using clinically relevant predictors, irrespective of their statistical significance in univariable analyses. Age, sex, Child–Pugh class, and etiology (alcohol-related vs. non-alcohol-related) were entered simultaneously into the model. Patient-reported variables derived from the SF-LDQOL questionnaire, including limitation of daily activities, liver symptom burden, and social isolation, were not included as predictors in the primary multivariable model in order to avoid conceptual overlap with the patient-reported quality-of-life outcome. Child–Pugh class was retained as the principal integrated measure of liver disease severity because it was systematically available for all participants and incorporates both laboratory and clinical components of hepatic dysfunction. MELD scores were not systematically calculated as part of the original study protocol and were therefore not included in the analysis. Individual Child–Pugh components were not entered simultaneously with Child–Pugh class in the multivariable model in order to avoid redundancy and unnecessary model complexity.

Overall quality of life was categorized as preserved (excellent/good) versus impaired (fair/poor), providing an analytically interpretable distinction between favorable and unfavorable self-perceived quality of life. This categorization represents an analytical grouping rather than a formally validated clinical cut-off. Because the original outcome comprised four ordered categories rather than a continuous score, treating it as a continuous variable would require an assumption of equal spacing between categories that could not be justified.

An ordinal logistic regression using the four original ordered quality-of-life categories was additionally evaluated. However, the marked concentration of poor quality-of-life ratings among patients in Child–Pugh classes B and C resulted in quasi-complete separation and unstable parameter estimates for Child–Pugh class. Because separation was also present in the binary outcome model, penalized logistic regression using Firth’s bias-reduction approach was used for the primary multivariable analysis to obtain finite and more stable parameter estimates. Multicollinearity among predictors included in the multivariable model was assessed using variance inflation factors (VIFs) and tolerance values.

The results are presented as odds ratios (ORs) with corresponding 95% confidence intervals (95% CIs). Statistical significance was defined as a two-sided *p*-value < 0.05. Both statistical significance and the magnitude of the observed associations were considered when interpreting the results in order to better assess their potential clinical relevance. Statistical analyses were performed and verified using Python version 3.13.5, with pandas version 2.2.3, NumPy version 2.3.5, SciPy version 1.17.0, and statsmodels version 0.14.6. Firth penalized logistic regression was implemented using the bias-reduced penalized-likelihood approach with numerical routines available in NumPy and SciPy.

## 3. Results

### 3.1. Baseline Characteristics

The study included 144 adult patients diagnosed with liver cirrhosis. The sex distribution showed a predominance of males, with 119 men (82.6%) and 25 women (17.4%). The mean age of the patients was 52.5 ± 5.1 years.

Regarding etiology, alcohol-related cirrhosis was the predominant etiology, accounting for 50.0% of cases. Other etiologies included toxic non-alcoholic causes in 33 patients (22.9%), hepatitis C virus infection in 14 cases (9.7%), hepatitis B virus infection in 10 cases (6.9%), metabolic causes in 10 patients (6.9%), and HBV + HDV coinfection in 5 patients (3.5%) (Table 1).

The study population consisted predominantly of middle-aged male patients. Patients with diverse etiologies were included, with alcohol-related cirrhosis representing the largest individual etiological subgroup. The predominance of compensated cirrhosis provided an opportunity to evaluate health-related quality of life before the onset of advanced hepatic decompensation (Figure 1).

### 3.2. Quality-of-Life Profile

Assessment of health-related quality of life using the SF-LDQOL questionnaire showed that most patients perceived their overall quality of life as fair, whereas nearly one quarter rated it as poor. This indicates a significant variability in the subjective perception of disease impact on daily life (Table 2 and Figure 2).

Overall, the SF-LDQOL questionnaire demonstrated that most patients perceived their health status as fair or poor, despite the predominance of compensated liver disease. The majority also reported either moderate or marked limitations in daily activities, while liver-related symptoms were considered to have a moderate or major impact on everyday functioning. These findings suggest that subjective disease burden may be substantial even in the absence of severe clinical decompensation.

### 3.3. Correlation Analyses

Correlation analysis identified a weak negative correlation between the impact of liver-related symptoms on daily activities and overall quality of life (Spearman r = −0.186, *p* = 0.025). Although statistically significant, the magnitude of this association was small and should therefore be interpreted cautiously.

No statistically significant correlation was observed between fatigue and overall quality of life (Spearman r = −0.03, *p* = 0.71). This finding indicates that no meaningful monotonic association between these variables was detected in the present cohort, rather than demonstrating an absence of a potential relationship.

Additional analyses showed a moderate negative correlation between limitation of daily activities and overall quality of life (r = −0.41, *p* < 0.001), while social isolation showed a weak-to-moderate negative correlation with life satisfaction (r = −0.36, *p* < 0.001). Pain showed a weak-to-moderate positive correlation with limitation of daily activities (r = 0.33, *p* < 0.001), and fatigue showed a weak negative correlation with perceived energy levels (r = −0.29, *p* = 0.002) (Table 3).

The largest correlation observed in the present analysis was between limitation of daily activities and overall quality of life (r = −0.41), representing a moderate association. The remaining statistically significant correlations were weak to moderate in magnitude. In particular, although the association between liver-related symptom burden and overall quality of life reached statistical significance, its small magnitude (r = −0.186) indicates limited explanatory strength when considered in isolation. These findings were therefore interpreted according to both effect size and statistical significance and should not be considered evidence of causal relationships.

### 3.4. Relationship Between Child–Pugh Class and Quality of Life

Most patients were classified as Child–Pugh A (75.7%), followed by Child–Pugh B (17.4%) and Child–Pugh C (6.9%). Overall quality of life differed significantly across Child–Pugh classes (Kruskal–Wallis H = 89.02, *p* < 0.001). Posthoc pairwise comparisons using Dunn’s test with Bonferroni correction showed significant differences between Child–Pugh A and B (adjusted *p* < 0.001) and between Child–Pugh A and C (adjusted *p* < 0.001), whereas no significant difference was observed between Child–Pugh B and C. These findings indicate that the overall group difference was primarily driven by poorer quality-of-life ratings in Child–Pugh B and C compared with Child–Pugh A(Table 4).

In a subgroup analysis stratified by etiology, the association between Child–Pugh class and overall quality of life remained statistically significant in both patients with alcohol-related cirrhosis (*n* = 72; Kruskal–Wallis H = 35.47, *p* < 0.001) and those with non-alcohol-related cirrhosis (*n* = 72; H = 50.50, *p* < 0.001). In both etiological groups, more advanced Child–Pugh classes were associated with poorer overall quality-of-life ratings.

Overall comparison: Kruskal–Wallis H = 89.02, *p* < 0.001. Dunn’s posthoc pairwise comparisons with Bonferroni correction: A vs. B, adjusted *p* < 0.001; A vs. C, adjusted *p* < 0.001; B vs. C, not statistically significant.

The comparison across Child–Pugh classes demonstrated poorer perceived quality of life among patients with more advanced liver disease, with significant differences between Child–Pugh A and both Child–Pugh B and C. However, no statistically significant difference was detected between classes B and C. These findings suggest that patient-reported quality of life differs substantially between compensated and more advanced disease, while also emphasizing the complementary information provided by patient-reported outcomes alongside conventional clinical severity assessment.

### 3.5. Clinical and Demographic Factors Associated with Impaired Overall Quality of Life

To evaluate clinical and demographic factors associated with impaired overall quality oflife, a forced-entry multivariable model was fitted including age, sex, Child–Pugh class, and etiology (alcohol-related vs. non-alcohol-related), irrespective of their statistical significance in univariable analyses. Questionnaire-derived patient-reported variables were not included in the primary multivariable model in order to avoid conceptual overlap with the overall quality-of-life outcome.

Because all patients classified as Child–Pugh B or C had impaired overall quality of life, the data exhibited quasi-complete separation with respect to Child–Pugh class. Therefore, Firth penalized logistic regression was used to obtain finite and more stable parameter estimates.

After simultaneous adjustment for age, sex, Child–Pugh class, and etiology, Child–Pugh B was significantly associated with higher odds of impaired overall quality of life compared with Child–Pugh A (adjusted OR 33.32, 95% CI 1.90–582.94, *p* = 0.016). The estimate for Child–Pugh C versus A was also elevated, but did not reach statistical significance and was imprecisely estimated (adjusted OR 11.56, 95% CI 0.60–221.43, *p* = 0.104). Age, sex, and alcohol-related etiology were not independently associated with impaired overall quality of life in the adjusted model. The wide confidence intervals for the Child–Pugh estimates reflect the sparse outcome distribution and complete absence of preserved quality-of-life ratings among patients in Child–Pugh classes B and C; therefore, the magnitude of these associations should be interpreted cautiously. No evidence of problematic multicollinearity was identified among the predictors included in the revised multivariable model (VIF range: 1.018–1.105; tolerance range: 0.905–0.982) (Table 5).

The revised multivariable analysis indicates that clinical liver disease severity, rather than age, sex, or etiology, was the principal factor associated with impaired overall quality of life in this cohort. However, because of the sparse distribution of preserved quality-of-life outcomes in the more advanced Child–Pugh classes and the resulting wide confidence intervals, these estimates should be interpreted cautiously.

## 4. Discussion

The present study evaluated the clinical and functional impact of liver cirrhosis through a multidimensional assessment integrating disease severity, patient-reported symptoms and health-related quality of life. The findings indicate that the burden of cirrhosis extends beyond conventional clinical parameters, encompassing functional and psychosocial dimensions that are associated with patients’ perceived health status. Notably, in the revised multivariable clinical model, greater liver disease severity, particularly Child–Pugh B compared with Child–Pugh A, was associated with higher odds of impaired overall quality of life, whereas age, sex, and etiology were not independently associated with the outcome. At the same time, the observed associations between patient-reported functional and psychosocial measures and perceived health status support the complementary value of multidimensional patient-reported assessment.

Assessment of health-related quality of life has become an essential component in the comprehensive evaluation of patients with liver cirrhosis, complementing traditional clinical and biochemical indicators. Increasing evidence suggests that patient-reported outcomes capture dimensions of disease burden that are not fully reflected by conventional severity scores, particularly regarding physical functioning, emotional well-being and social participation. In the present study, associations between patient-reported functional and psychosocial measures and perceived health status support this multidimensional perspective and emphasize that these dimensions should be considered alongside clinical staging when evaluating the overall impact of liver cirrhosis [6,7,8].

In our cohort, the majority of patients were classified as Child–Pugh A, indicating predominantly compensated liver disease. Nevertheless, a substantial proportion reported only fair or poor health-related quality of life, suggesting that patients’ perceived health status may be considerably impaired even during the compensated stage of cirrhosis. These findings reinforce the concept that clinical compensation does not necessarily translate into preserved functional status and highlight the value of integrating patient-reported outcomes into the overall clinical assessment of liver cirrhosis.

The predominance of compensated cirrhosis in the present cohort deserves particular attention. Although patients with Child–Pugh A disease are generally considered clinically stable, accumulating evidence indicates that significant impairment in health-related quality of life may already be present at this stage. Fatigue, reduced physical performance, impaired concentration, sleep disturbances and psychological distress frequently precede overt hepatic decompensation, adversely affecting patients’ daily functioning and perceived health status. Our findings are consistent with these observations and further suggest that conventional clinical staging alone may not fully capture the multidimensional impact of liver cirrhosis. This concept is further supported by the associations observed between patient-reported functional and psychosocial measures and perceived health status, reinforcing the need for comprehensive patient-centred assessment alongside traditional clinical evaluation [12].

The predominance of alcohol-related cirrhosis in our cohort is consistent with epidemiological data from Eastern Europe, where alcohol remains the leading etiological factor for advanced chronic liver disease [3,13]. Beyond its direct hepatic effects, alcohol-related cirrhosis is frequently accompanied by nutritional deficiencies, sarcopenia, anxiety, depression and increased social vulnerability, all of which contribute to a multidimensional disease burden that may not be fully reflected by conventional clinical severity scores [14]. In our study, the observed associations between psychosocial measures and patient-reported health outcomes further suggest that psychosocial dimensions represent an important component of patients’ perceived health status. Consequently, the management of alcohol-related cirrhosis should extend beyond the treatment of liver dysfunction alone, incorporating nutritional support, psychological assessment, physical rehabilitation and social interventions within a multidisciplinary care model whenever appropriate [15].

Our results showed that liver-related symptom burden was statistically correlated with overall health-related quality of life; however, the magnitude of this association was small (r = −0.186), indicating limited explanatory strength when considered in isolation. Limitation of daily activities showed a moderate correlation with overall quality of life (r = −0.41), indicating a stronger association than that observed for liver-related symptom burden. These findings suggest that statistically significant correlations should be interpreted in the context of their effect size and support a multidimensional understanding of health-related quality of life, in which clinical, functional, and psychosocial factors may contribute simultaneously to patients’ perceived health status. Patient-reported symptoms and functional limitations may therefore provide complementary information alongside conventional clinical assessment.

The observed relationship between psychosocial factors and patient-reported health outcomes represents a clinically relevant aspect of the present study. In particular, social isolation was significantly associated with poorer patient-reported outcomes in the exploratory analyses, reinforcing growing evidence that health-related quality of life in liver cirrhosis is associated not only with physical symptoms but also with psychological and social dimensions [16,17,18,19]. Anxiety, depression, reduced social support, financial difficulties and stigma associated with chronic liver disease may adversely affect patients’ perceived health status [20]. Taken together, these findings support a patient-centred approach to the management of liver cirrhosis, in which psychosocial assessment may complement routine clinical evaluation to identify supportive care needs and facilitate individualized multidisciplinary care.

Interestingly, fatigue was not significantly correlated with overall health-related quality of life in the present cohort (r = −0.03, *p* = 0.71), although it showed a significant negative correlation with perceived energy levels (r = −0.29, *p* = 0.002). The absence of a statistically significant correlation with overall quality of life should not be interpreted as evidence that fatigue has no impact on patients’ well-being. This finding should be interpreted cautiously and does not imply that fatigue is clinically irrelevant in patients with cirrhosis. One possible explanation is that overall quality of life represents a multidimensional construct influenced by several functional, clinical, and psychosocial factors, such that the specific association with fatigue may not be detectable when assessed against a global quality-of-life measure. In support of this interpretation, fatigue was significantly associated with lower perceived energy levels in our cohort (r = −0.29, *p* = 0.002), suggesting that its relationship with health status may be more readily captured within specific health-related quality-of-life dimensions than by the global quality-of-life assessment. Rather, this finding may reflect the complex and multifactorial nature of fatigue in chronic liver disease, whose effects may be more apparent within specific functional domains than in a global quality-of-life measure [21,22].

A statistically significant difference in health-related quality of life was observed across Child–Pugh classes. Posthoc pairwise analyses showed significantly poorer quality-of-life ratings in Child–Pugh B and C compared with Child–Pugh A, whereas no statistically significant difference was detected between Child–Pugh B and C. Thus, although quality of life was less favorable in patients with more advanced disease than in those with Child–Pugh A cirrhosis, the findings did not demonstrate a strictly progressive gradient across all three Child–Pugh classes. This pattern is consistent with previous evidence indicating an association between liver disease severity and patient-reported quality of life [6,23,24]. Moreover, the observation that impaired quality of life was also present among patients with compensated cirrhosis suggests that conventional clinical staging alone does not fully capture patients’ perceived health status. At the same time, the revised multivariable clinical analysis identified Child–Pugh B as significantly associated with higher odds of impaired overall quality of life compared with Child–Pugh A, whereas the estimate for Child–Pugh C was also elevated but imprecise and did not reach statistical significance. The wide confidence intervals observed for the more advanced Child–Pugh classes warrant cautious interpretation. Together with the observed associations between patient-reported functional and psychosocial measures and perceived health status, these findings support the complementary role of clinical disease severity and patient-reported assessment in characterizing the overall burden of cirrhosis [18,25,26].

These findings are consistent with previous studies demonstrating that impairment in health-related quality of life may occur early during the course of chronic liver disease, even in clinically compensated patients [6,9]. Younossi et al. reported that patients with chronic liver disease frequently experience reduced quality of life despite preserved liver function [27], while studies conducted in Asian cohorts similarly demonstrated that both disease severity and psychosocial factors contribute to patients’ perceived health status [28,29]. Our findings extend these observations by showing that liver disease severity remained relevant to impaired overall quality of life after simultaneous adjustment for age, sex, and etiology, while patient-reported functional and psychosocial measures provided complementary information regarding patients’ perceived health status.Taken together, these results support the concept that conventional prognostic classifications and patient-reported outcome measures should be regarded as complementary rather than competing approaches. While prognostic scores remain indispensable for assessing disease severity, patient-reported outcomes provide additional insight into the functional and psychosocial consequences of liver cirrhosis, thereby supporting more individualized clinical assessment and multidisciplinary patient management.

An additional aspect that deserves consideration is the growing recognition of impaired physical performance and frailty as clinically relevant manifestations of chronic liver disease. Although these parameters were not specifically assessed in the present study, recent evidence indicates that reduced physical function is closely associated with poorer health-related quality of life, decreased exercise tolerance, increased hospitalization and reduced survival [30]. In the context of our findings, these observations further support the concept that the clinical impact of liver cirrhosis extends beyond hepatic dysfunction alone. Consequently, integrating conventional prognostic scores with patient-reported outcome measures and functional assessment may provide a more comprehensive evaluation of patients and facilitate individualized multidisciplinary management.

From a clinical perspective, these findings have important implications. The assessment of patients with liver cirrhosis should not rely exclusively on biological markers or prognostic scores such as the Child–Pugh classification [26]. Patient-reported outcomes, symptom burden and functional status provide complementary information regarding the overall impact of the disease and should be integrated into routine clinical evaluation. Such a multidimensional approach may facilitate more individualized patient management, improve the identification of unmet supportive care needs and contribute to comprehensive multidisciplinary care [31,32,33].

Taken together, the present findings suggest that health-related quality of life in liver cirrhosis is associated with multiple clinical, functional and psychosocial factors, including disease severity, symptom burden, functional limitations and psychosocial well-being. While conventional prognostic scores remain fundamental for staging liver disease, patient-reported outcome measures provide complementary information regarding the broader impact of liver cirrhosis on patients’ everyday lives. Integrating these dimensions into routine clinical assessment may contribute to a more comprehensive understanding of patients’ needs and support individualized multidisciplinary management.

The present findings further support the growing role of patient-reported outcome measures in the comprehensive evaluation of patients with chronic liver disease. Beyond conventional biochemical and prognostic parameters, these instruments provide valuable information regarding symptom burden, functional status and patients’ perceived health, thereby complementing routine clinical assessment. Such an integrated approach may facilitate more individualized patient management and help identify supportive care needs that may not be apparent through traditional clinical evaluation alone [34].

From a practical perspective, incorporating standardized health-related quality-of-life assessment into the routine evaluation of patients with liver cirrhosis may facilitate the early identification of functional limitations and supportive care needs. Combined with conventional clinical assessment, this approach may contribute to more individualized multidisciplinary management aimed at preserving patients’ health-related quality of life and overall functional status [35]. The broader spectrum of chronic liver disease also encompasses major complications such as hepatocellular carcinoma, for which ongoing research continues to identify novel molecular pathways and potential biomarkers. Recent experimental and bioinformatics studies, including investigations of lumican in hepatocellular carcinoma, further illustrate the biological complexity of advanced liver disease and the expanding multidimensional framework within which chronic liver disorders are currently investigated [36].

The present study has several limitations. First, its cross-sectional design precludes the assessment of temporal relationships and causal effects; therefore, the associations identified between clinical, functional, psychosocial factors and health-related quality of life should not be interpreted as evidence of causality. Second, participants were recruited from a specialized national institute for medical expertise and work-capacity assessment rather than from an unselected hepatology population. This specific recruitment setting may have preferentially included patients with greater functional impairment or work-related consequences of their disease, introducing potential selection bias and limiting the external generalizability of the findings. In addition, the predominance of patients with compensated cirrhosis may limit the applicability of the results to individuals with more advanced disease. Finally, although patient-reported outcome measures provide valuable insight into patients’ perceptions of health, responses may be influenced by individual psychological and social factors. Moreover, potentially relevant psychological confounders, particularly depression and anxiety, were not assessed using dedicated validated instruments and therefore could not be included as covariates in the multivariable model. Given the potential relationships between psychological distress, psychosocial functioning, and perceived quality of life, residual confounding cannot be excluded when interpreting the observed associations between patient-reported psychosocial measures and health-related quality of life. Future longitudinal, multicenter studies involving more diverse cirrhosis populations and incorporating validated measures of depression, anxiety, and other psychosocial factors are needed to further clarify the relationships between clinical, functional, and psychosocial factors and health-related quality of life.

The present study also has several strengths. It included a relatively large cohort of consecutively evaluated patients with liver cirrhosis, enhancing the clinical relevance of the findings. Furthermore, the simultaneous assessment of disease severity, symptom burden, functional status and patient-reported outcomes enabled a comprehensive multidimensional evaluation of the impact of cirrhosis. Finally, the revised multivariable analysis incorporated pre-specified clinical and demographic variables and demonstrated an association between liver disease severity and impaired overall quality of life, while the complementary patient-reported analyses provided additional insight into the functional and psychosocial dimensions of the disease burden.

## 5. Conclusions

Liver cirrhosis is associated with substantial impairment in health-related quality of life. Greater liver disease severity was associated with poorer quality of life, including in the revised multivariable clinical analysis, although impaired health-related quality of life was also observed in a considerable proportion of patients with compensated cirrhosis. The present findings further indicate that symptom burden, functional limitations, and psychosocial factors are associated with patients’ perceived health status, emphasizing the complementary value of integrating patient-reported outcome measures with conventional clinical assessment. Such a multidimensional approach may facilitate a more comprehensive evaluation of disease burden, support individualized multidisciplinary management, and promote patient-centered care in individuals with liver cirrhosis.

## Figures and Tables

**Figure 1:**
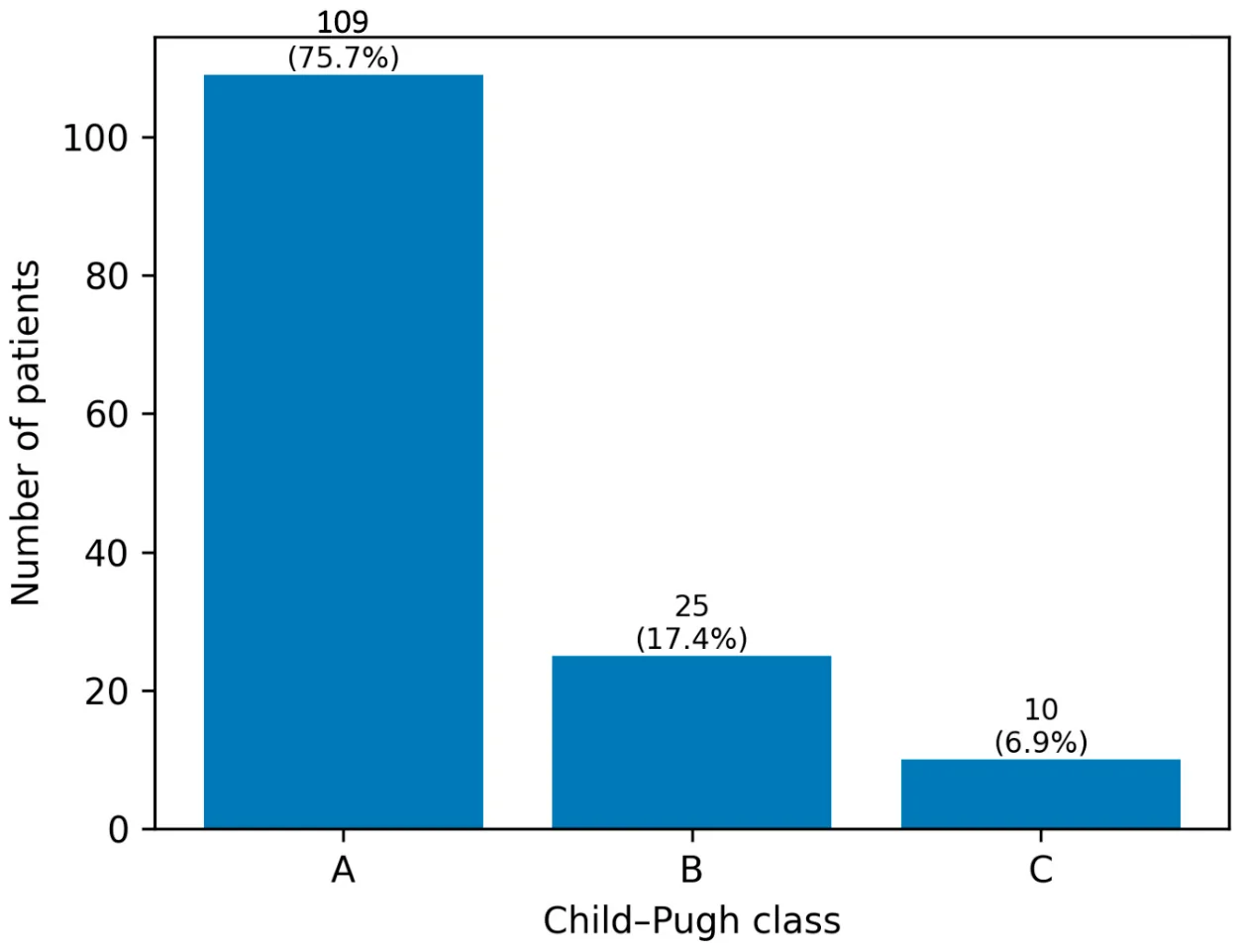
Distribution of the study population according to Child–Pugh classification (*n* = 144). Most patients were classified as Child–Pugh A (75.7%), followed by Child–Pugh B (17.4%) and Child–Pugh C (6.9%), indicating a predominance of compensated liver disease.

**Figure 2:**
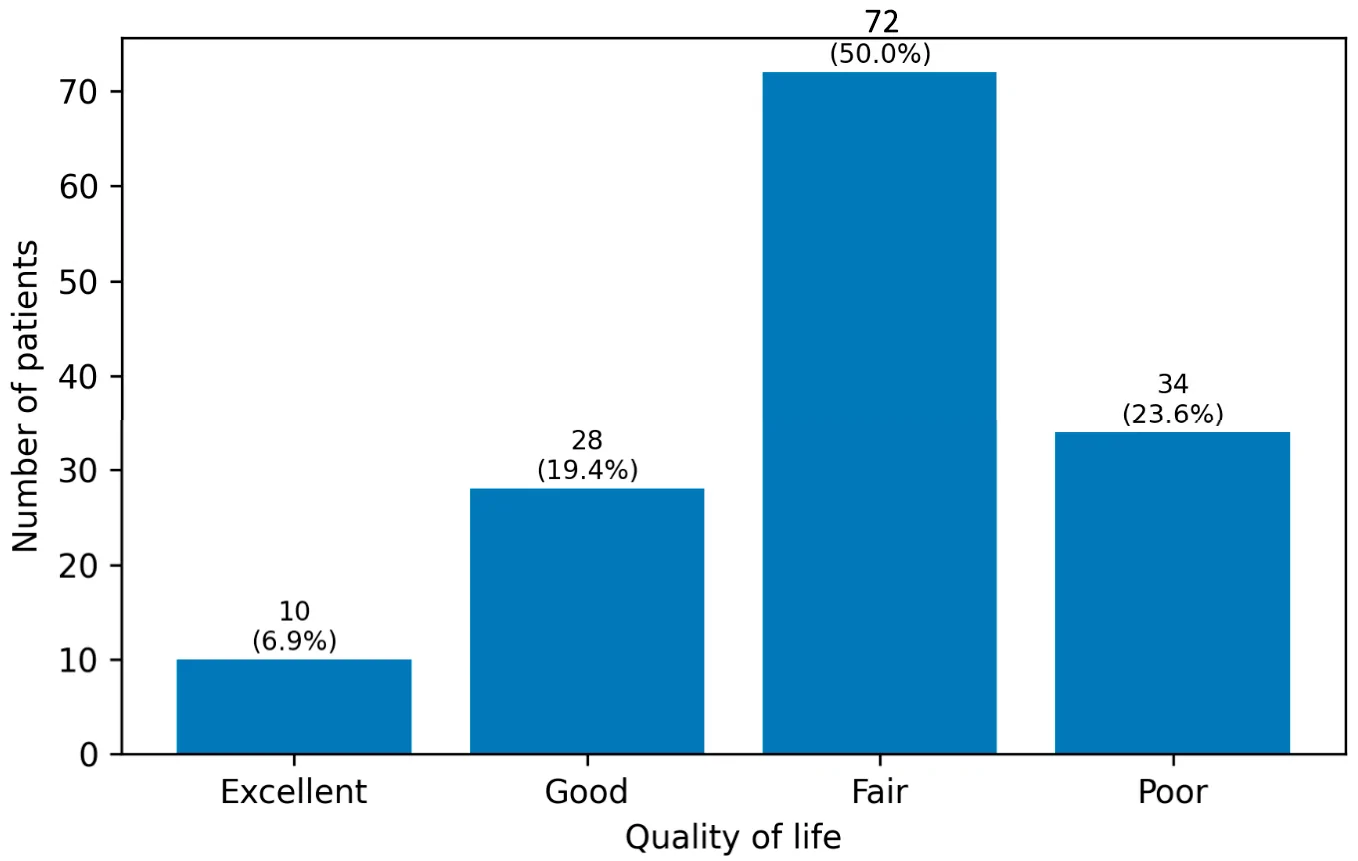
Distribution of patients according to their self-reported overall quality of life (*n* = 144). Fair quality of life was the most frequently reported category (50.0%), followed by poor (23.6%), good (19.4%), and excellent (6.9%).

**Table 1:** Baseline characteristics of the study population.

Variable	Value
Number of patients	144
Age (years), mean ± SD	52.5 ± 5.1
Sex	
Male	119 (82.6%)
Female	25 (17.4%)
Etiology of liver cirrhosis	
Alcohol-related	72 (50.0%)
Toxic (non-alcoholic)	33 (22.9%)
Hepatitis C virus (HCV)	14 (9.7%)
Hepatitis B virus (HBV)	10 (6.9%)
Metabolic	10 (6.9%)
HBV + HDV coinfection	5 (3.5%)

**Table 2:** Quality of life assessment based on the SF-LDQOL questionnaire.

Variable	*n* (%)
Self-rated general health	
Good	18 (12.5%)
Fair	64 (44.4%)
Poor	33 (22.9%)
Very poor	29 (20.1%)
Health compared with previous year	
Much better	4 (2.8%)
Slightly better	14 (9.7%)
About the same	48 (33.3%)
Slightly worse	50 (34.7%)
Much worse	28 (19.4%)
Limitation of daily activities	
None	14 (9.7%)
Slight	29 (20.1%)
Moderate	42 (29.2%)
Much	49 (34.0%)
Very much	10 (6.9%)
Impact of liver symptoms on daily activity	
None	14 (9.7%)
Slight	10 (6.9%)
Moderate	57 (39.6%)
Much	25 (17.4%)
Very much	38 (26.4%)
Overall quality of life	
Excellent	10 (6.9%)
Good	28 (19.4%)
Fair	72 (50.0%)
Poor	34 (23.6%)

**Table 3:** Correlation between clinical symptoms and quality of life indicators.

Variables	Spearman r	*p* Value
Liver symptoms impact ↔ Overall quality of life	−0.186	0.025
Limitation of daily activities ↔ Overall quality of life	−0.41	<0.001
Social isolation ↔ Life satisfaction	−0.36	<0.001
Pain ↔ Limitation of daily activities	0.33	<0.001
Fatigue ↔ Energy level	−0.29	0.002

**Table 4:** Overall quality of life according to Child–Pugh class.

Child-Pugh Class	Number of Patients	Overall Quality of Life (Predominant Perception)
A	109 (75.7%)	Fair/Good
B	25 (17.4%)	Fair/Poor
C	10 (6.9%)	Poor

**Table 5:** Forced-entry Firth penalized logistic regression analysis of clinical and demographic factors associated with impaired overall quality of life.

Variable	Adjusted OR (95% CI)	*p*-Value
Age (per year)	0.98 (0.90–1.06)	0.587
Male sex	0.93 (0.33–2.63)	0.898
Child–Pugh B vs. A	33.32 (1.90–582.94)	0.016
Child–Pugh C vs. A	11.56 (0.60–221.43)	0.104
Alcohol-related vs. non-alcohol-related etiology	1.75 (0.78–3.88)	0.172

Abbreviations: OR, odds ratio; CI, confidence interval. Reference categories: female sex, Child–Pugh A, and non-alcohol-related etiology.

## Data Availability

The data presented in this study are available on request from the corresponding author due to legal and ethical reasons.
